# Medical students describe their wellness and how to preserve it

**DOI:** 10.1186/s12909-022-03552-y

**Published:** 2022-06-28

**Authors:** Krishanu Chatterjee, Victoria S. Edmonds, Marlene E. Girardo, Kristin S. Vickers, Julie C. Hathaway, Cynthia M. Stonnington

**Affiliations:** 1 Mayo Clinic Alix School of Medicine, Mayo Clinic, Scottsdale, AZ USA; 2 Department of Psychiatry, University of Illinois at Chicago, Chicago, IL USA; 3 Department of Urology, Mayo Clinic, AZ Phoenix, USA; 4 Department of Quantitative Heath Sciences, Mayo Clinic, Phoenix, AZ USA; 5 Department of Psychiatry & Psychology, Mayo Clinic, Rochester, MN USA; 6 Office of Patient Education Research, Mayo Clinic College of Medicine and Science, Rochester, MN USA; 7 Department of Psychiatry and Psychology, Mayo Clinic, Scottsdale, AZ USA

**Keywords:** Education environment, Qualitative analysis, Undergraduate medical education, Student wellness, Wellness curriculum

## Abstract

**Background:**

Despite widespread efforts to create wellness programming in medical schools, there is a paucity of literature examining students’ perception of wellness and perceptions of these programs. With the inaugural class at the Arizona campus of Mayo Clinic Alix School of Medicine (MCASOM-AZ), an opportunity arose to establish an empirically evaluated wellness curriculum that most inclusively and effectively enables medical students to flourish for years to come. The initial wellness offerings included mental health, academic success, and disability services, curriculum-embedded seminars, wellness committee driven programming, and student-proposed wellness activities. We aimed to improve the relevance and impact of medical school wellness curricula by soliciting in-depth and longitudinal perspectives of medical students themselves. As MCASOM-AZ opened in 2017, the student body at the time of study consisted of first- and second-year medical students.

**Methods:**

Employing a mixed methods analysis of qualitative and longitudinal quantitative data, first- and second-year students at a MCASOM-AZ were invited to respond to an anonymous, online year-long survey (baseline, six months and 12 months) during the 2018–2019 academic year and participate in a structured, in-depth and in-person, peer-to-peer interview about their conceptions of wellness and the MCASOM-AZ wellness curriculum and resources. Qualitative data was coded for themes using thematic analysis strategies by independent raters.

**Results:**

Nearly half of eligible students completed the baseline survey,1/3 completed all 3 time-points, and 1/5 participated in an in-depth interview. Participant age, gender, and year of school were representative of the larger student body. Although individual conceptions varied, Wellness was consistently highly valued. Family, Academic Performance, and Friends emerged as most important to well-being across time-points. Academic work arose as the largest barrier to wellness. Analysis of qualitative data revealed five themes. Despite individual differences in approaches to wellness, wellbeing was interrelated to the learning environment; mandatory wellness efforts that didn’t address the medical culture met with skepticism.

**Conclusions:**

Interview responses provided understanding and context by which to interpret questionnaire responses. Academics was critical to students’ identity and wellness, while also the largest barrier. Suggested curricular improvements include restructuring academic work, seamlessly integrating wellness within coursework, and offering optional individualized approaches.

**Supplementary Information:**

The online version contains supplementary material available at 10.1186/s12909-022-03552-y.

## Background

As defined by The World Health Organization, wellbeing is “a positive state experienced by individuals and societies” and wellness is “a state of complete physical, mental, and social well-being, and not merely the absence of disease or infirmity.” In recent years, student wellness during medical school has garnered much needed attention. The increased awareness of physician burnout and depression [1, 2], which has been shown to begin in medical school, [3] motivated individual medical schools as well as the governing bodies of the American Medical Association and the Association of American Medical Colleges to launch programs to assess levels of distress in medical students [4, 5] and to identify the specific stressors responsible [6–8]. The pressure to score highly on examinations, stigma around help-seeking, and a surrounding culture that prioritizes performance over wellness have been a few of the drivers implicated. [9, 10]. These findings have cumulatively led to curricular changes in medical schools throughout the country such as pass/fail grading and integration of wellness programs [11], as well as the recognition that maintaining wellness ought to be a core physician competency [12]. Despite the paucity in empirically evaluated interventions [13], the promise of fostering students’ resilience and professional development via expanded wellness programs at medical school is compelling [11].

The input of medical students themselves has been recognized as crucial to the development and employment of these changes [14, 15]. Collection of qualitative data from students is one way to gather such insights. In previous studies, medical students were recruited to identify their most salient stressors as well as the self-care behaviors they use to cope with these stressors [16, 17] in order to build the framework for curricular change [18]. Similarly, in other medical school populations, written responses were gathered to open-ended questions regarding the stressors and barriers to wellness encountered in medical school [18–20]. Another compelling study gathered verbal responses to a wide-ranging set of questions about their entire medical school experience through “life-story interviews” with medical students who had already undergone the National Residents Matching Program match [21]. None of these studies, however, sought a mixed methodology, where the details identified through qualitative means could be correlated with longitudinal quantitative survey measures among the same cohort. Such mixed methodology adds context and nuance to the quantitative data and thus may help to understand why some well-intentioned programs or curricular changes succeed or fail. Furthermore, a thorough exploration of how students personally conceptualize the wellness that is at stake in this topic appears lacking in the present literature. Since the topic of wellness is inherently subjective and driven by unique personal values, there is a challenge to align curricular changes with these diversely-held attitudes and approaches towards personal wellness.

With the inaugural class at the Arizona campus of Mayo Clinic Alix School of Medicine (MCASOM-AZ), an opportunity arose to refine a wellness curriculum that is optimally valued by its students. Our long-term goal was to establish an empirically evaluated wellness curriculum that most inclusively and effectively enables students to flourish during medical school for years to come. To this end, first and second year student perspectives on wellness, including their individual definitions of wellness, strategies for maintaining wellness, barriers to wellness, and attitudes about the existing wellness curriculum, were gathered qualitatively in the form of sit-down interviews conducted by their peers. This was in addition to surveys that assessed repeated categorical and validated measures of student wellness at three timepoints over one year. We recently reported on some of the data from this study [22]. That report detailed the initial wellness curriculum and results of the categorical, empirically validated survey data, which showed significant increase in wellbeing (as measured by the World Health Organization Wellbeing Index) and decrease in perceived stress (as measured by the Perceived Stress Scale) over the course of the year, with no difference between students who participated in the wellness curriculum and those who didn’t and no association between age or gender and measured wellbeing or perceived stress at any time point. [22]. Furthermore, students perceived unscheduled time as being most impactful to their wellness and student-initiated activities as second-most impactful [22].

This report focuses on the interview data and student rankings of factors associated with wellness. We aimed to uncover key themes in how medical students describe and maintain their wellness, their solutions to perceived barriers, why they preferred unstructured time or student-led curriculum to faculty-led wellness curriculum, and to further understand the reasons for our findings in the quantitative and longitudinal data reflecting changing preferences in activities that helped to maintain wellness. We employed a mixed methods approach of gathering verbal, qualitative responses to relevant prompts anchored on individual conceptualizations of wellness, and cross-analyzing this data with responses to related questions over time among the same student cohort. Importantly, the administration of the interviews and surveys were conducted by peers, allowing a level of trust and collegiality that is crucial for gathering honest responses. The insights from this study helped refine our wellness programing, and we expect our approach and findings to inform other medical schools with their curricula and wellness programming.

## Methods

### Participant recruitment

At the time of this study, MCASOM-AZ had been open for two years, so all recruited participants were either year 1 or 2 students. Each year, MCASOM-AZ matriculates 50 students. All 100 MCASOM-AZ students were asked to participate in both the quantitative (survey-based) and qualitative (interview-based) components of this study, and of these 49 agreed to participate. Participation was elective and all responses were anonymous. The Mayo Clinic Institutional Review Board and the Mayo Clinic College of Medicine and Science Education Research Committee approved this study (protocol number 18–005,605). We informed participants that participation was voluntary and that their current and future employment, education, and medical care at Mayo Clinic would not be affected by whether or not they participated. Participants received a $20 incentive for completing all three surveys, or for completing the in-person interview.

### Quantitative data collection

We surveyed participants electronically at three points during the 2018–2019 academic year. We distributed a “baseline” survey in July 2018, a “midterm” 6 month follow-up survey in January 2019, and a “final” 1 year follow-up survey in June 2019. We explained to participants via email that the purpose of the survey was to ask students about their perceived wellness and personal beliefs and attitudes towards wellness in order to develop a more effective and inclusive wellness curriculum.

Participants generated an anonymous unique identifying code that was used to link their data over the course of the study. Questionnaires were distributed at all three time points (available as Supplemental Digital Appendix 1). We refrained from asking demographic details other than gender and age group to maintain anonymity given the small student body. Students were first asked to rank 8 factors, based on prior literature, team discussion, and anecdotal student input, in order of most to least important to their overall wellness (academic performance, [23, 24] physical fitness, [25, 26] family, [27, 28] financial stability, [24] friends, [27, 28] hobbies, [29] alone time, [30] sleep [23, 26, 31]). Then, participants ranked 8 accomplishments to feeling successful as a medical student, ranging from publishing research and matching into a first-choice residency program to keeping up with friends and maintaining a healthy diet, in order of most to least important. Participants also were asked to rate, on a scale from 1 to 10, the importance they placed on maintaining wellness in medical school. Such single item ratings have been shown valid and clinically meaningful in other settings [32, 33]. Finally, they were asked to respond in free-text to the questions “What do you do to keep yourself feeling well?” and “What do you do when you are not feeling well?” and subsequently, “What gets in the way of you doing those things?” For the third question, participants selected one or more factors as barriers, based on prior literature, team discussion, and anecdotal student input, including schoolwork, family responsibilities, research, friends, finances, and physical or mental health.

Descriptive statistics were used to summarize participant questionnaire responses. Highly important ranked responses were compared to not important. Grouped responses for feeling well versus not feeling well were also compared. Categorical variables were compared using Chi-square test. *P*—values < 0.05 were considered significant. We conducted all analyses using SAS Version 9.4 (SAS Institute Inc.).

### Qualitative data collection

All 100 students were also invited for an in-person, extended interview, of whom a 20- student subset of the 49 who had consented and completed the first questionnaire agreed to schedule a mutually convenient time with one of the student investigators (KC). Interviews were held between October and December 2018, when first-year students had already been through rigorous early coursework including Human Anatomy, and second year students had begun their Organ-System based instruction. All interviews were conducted by the same student investigator. Participants were informed that the interview would be recorded, transcribed, and analyzed, and that all recorded data would be anonymized. All interviews were held in private rooms at the MCASOM-AZ campus. Participants were able to end the interview at any time with no consequence or loss of reward.

**Fig. 1:**
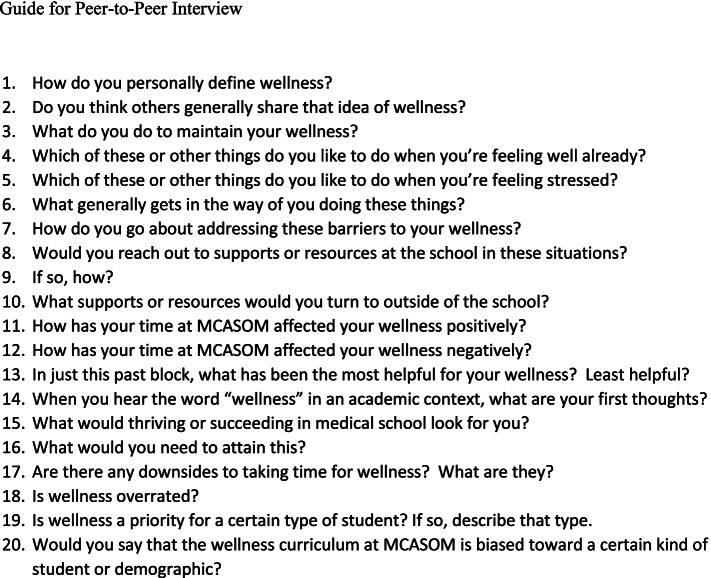
List of question prompts used to guide sit-down wellness interview

All interviews were guided by the same set of 20 question prompts, followed in order (Fig. 1). Similar to the quantitative survey, these interview prompts were generated by the investigators based on prior literature, [15, 16, 24, 27–29, 31, 34] team discussion, and anecdotal student input. Specifically, they were informed by literature on wellness that emphasized mental, emotional, social, spiritual, and financial health. We also considered self-determination theory, [34] which emphasizes autonomy, social relatedness, and competency as critical to well-being. However, we also wanted to be as open-ended as possible so as not to encourage any one type of response or orthodoxy. These prompts were asked with the intention to understand reasons for what we were seeing in the quantitative data and to stimulate conversation around the topic of student wellness, including: personal conceptions of wellness, strategies for maintaining wellness, perceived barriers to wellness, differences in behavior when already feeling well or unwell, the impact of MCASOM-AZ curriculum and resources on wellness, and the portrayal of wellness in medical education. Interviews were not limited in time, though anticipated to last 30–45 min.

Qualitative data was analyzed using methods of thematic analysis, which is defined as the systematic process of sorting information based on identified themes [35–38]. Dominant themes, based on multiple participants repeating similar experiences, attitudes, or needs, were identified and a coding strategy was developed. Three investigators (KV, JH, and KC) independently coded all qualitative data, compared categories and refined the parameters of each theme. Any discrepancies in coding to themes prompted review of full interview transcript to better understand meaning through examination of the original context. Aside from the student KC, two of the coding investigators (KV and JH) have experience in qualitative methods and were brought in to assist with analysis to address reliability and validity of coding and reporting data [35, 37]. The two non-student coders (KV and JH) were not involved with the medical school, study design, sampling approach, or data collection, and not involved in implementing wellness programming in the medical school. Consequently, they may be considered free of biases related to medical school students and wellness curriculum. KC, as a current medical student and as the primary interviewer, brought another perspective to the analysis process. The investigators practiced self-reflection and discussed potential for bias in coding and reporting themes. After these two investigators independently coded qualitative data, predominant theme categories were agreed upon and representative quotes were identified for each theme.

## Results

### Quantitative data analysis

#### Participants

58 of 100 students consented to participate however of those consented, only 49 went on to respond to the baseline questionnaire, 30 to the midterm questionnaire (six-month follow-up), and 29 to the final questionnaire (12-month follow-up). Year 1 and year 2 students were almost equally represented among those who consented to participate (41.4% and 58.6% respectively). Gender was almost equally represented throughout (at baseline 51% female, 49% male, none non-confirming). Age distribution is shown in Table 1. There was no difference in age (*X*^*2*^ (2, 49) = 3.68, *p* = 0.159) or gender (*X*^*2*^ (1, 49) = 0.008, *p* = 0.928) between participants who only responded at baseline and those who completed one or both follow-up questionnaires. However, as outlined in our prior report [22], participants who only completed the baseline questionnaire had significantly lower mean WHO-5 scores compared with those who completed one or both follow-up’s (12.9 vs. 15.8). In addition, the respondents in this study were representative of the student population as a whole based on age and gender.

**Table 1:** Demographic breakdown of respondents at baseline, midterm (6-month follow-up), and final (12-month follow-up) time-points

	**Baseline**	**Midterm**	**Final**	***P*-value**
**Age**, n (%)				0.1588^1^
≤ 23	21 (42.9%)	6 (20.0%)	6 (20.7%)	
24–29	22 (44.9%)	19 (63.3%)	19 (65.5%)	
≥ 30	6 (12.2%)	5 (16.7%)	4 (13.8%)	
**Gender**, n (%)				0.9280^1^
Female	25 (51.0%)	18 (60.0%)	14 (48.3%)	
Male	24 (49.0%)	12 (40.0%)	15 (51.7%)	
Non-conforming	0 (0%)	0 (0%)	0 (0%)	
**Total**, n	49	30	29	

^1^Chi-Square *p*-value. There was no difference in age or gender between participants who only responded at baseline and those who completed one or both follow-up questionnaires

#### Conceptions of wellness and success

**Fig. 2:**
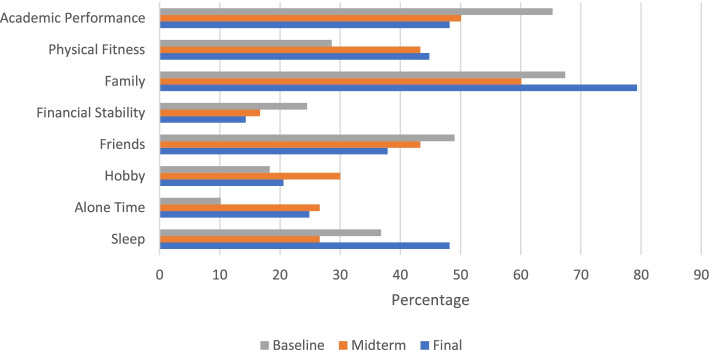
Importance Rankings of Wellness Factors. Preclinical students were asked to rank these 8 factors, in order of most to least important to their overall wellness. Percentage of students that ranked a given factor either 1, 2, or 3 is graphically shown for each timepoint (baseline, midterm (6 months), and final (12 months)

Results from the individual ranking of factors contributing to participants’ overall wellness are graphically shown in Fig. 2. Family was most important (i.e., ranked 1, 2, or 3) at both baseline (*n* = 33, 67.4%) and final (*n* = 23, 79.3%). While 32 (65.3%) students felt academic performance was most important at baseline, this decreased to 14 (48.2%) at final. Physical fitness increased in importance with 14 (28.6%) students selecting it as most important at baseline and 14 (44.8%) at final. Similarly, alone time increased in importance with 5 (10.2%) selecting it as most important at baseline and 7 (24.9%) at final. Financial stability was consistently ranked as least important (i.e., ranked 6, 7, or 8) to overall wellness with 23 (46.9%) at baseline and 16 (57.2%) at final.

The two areas that the majority of students felt were important (i.e., ranked 1 or 2) to their success as a medical student included doing well on board exams and matching into a first choice residency program. Interestingly, the percentage of students who ranked board exams as important decreased from 37 (75.5%) at baseline to 17 (58.6%) at final. Matching into a first choice residency program decreased, too, from 29 (59.2%) to 15 (51.7%). Spending time with family which was ranked at third place at baseline became more important to students’ success over the year from 24 (48.9%) to 16 (55.1%) at final.

Across all three time points, students who ranked academic performance as important to their overall wellness also selected doing well on board exams and matching into a first-choice residency program as important to feeling successful as a medical student. For instance, at baseline, of the 26 (53.1%) students who indicated academic performance as highly important to their overall wellness (ranked 1 or 2), 21 (80.8% vs 39.1%) selected doing well on board exams (*X*^*2*^ (1, 49) = 18.86, *p* < 0.001) and 17 (65.4% vs 30.4%) selected matching into a first-choice residency program (*X*^*2*^ (1, 49) = 12.15, *p* = 0.015) as highly important (ranked 1 or 2) to feeling successful as a medical student. Conversely, students who rated academic performance as less important to their overall wellness also rated academically-related accomplishments (board exams, first-choice residency program) less highly (not ranked 1 or 2) in whether or not they felt successful as a medical student. At baseline, of the 23 (46.9%) students who indicated academic performance was not as important to their overall wellness (not ranked 1 or 2), 19 (82.6%) felt publishing research was not highly important (not ranked 1 or 2) to their success as a medical student and 19 (82.6%) indicated the same for becoming involved in extracurricular activities, although these were not statistically significant.

We asked students to indicate how important it was to them to maintain their wellness while in medical school on a scale from 1 (not important) to 10 (most important). The majority of respondents felt this was highly important (9 or 10) at all three time points; 29 (59.2%) at baseline, 19 (63.4%) at midterm, and 16 (57.2%) at final. Students who ranked academic performance as highly important (1 or 2) to their overall wellness did not feel maintaining their wellness while in medical school was most important (10) as frequently as their peers (10 or 43.5% of whom ranked it 10). However, 23 (88.5%) still ranked it between 8 and 10.

#### Barriers to maintenance of wellness

Across our three time points across the academic year, we also queried students on whether work, family responsibilities, research, friends, finances, or physical and mental health were a barrier to engaging in activities they did to maintain their own wellness (i.e., their answers to free text questions below), with the option to select all that applied. Almost all respondents felt work was an obstacle with 44 (89.8%) at baseline, 29 (96.7%) at midterm, and 29 (100%) at final. A majority of respondents did not feel that family responsibilities, friends, finances, or their physical or mental health were an impedance in this regard at any of the timepoints.

## Qualitative data analysis

### Strategies for wellness & coping

Also on Questionnaire 1, we allowed students to respond in free-text to the questions “What do you do to keep yourself feeling well?” and “What do you do when you are not feeling well?” Students frequently referenced social activities, hobbies (reading, hiking, crafts), spiritual or religious engagements, exercise, diet, sleep, alone time, academic work, and mental health management (counseling and medications) in response to both questions. Students frequently mentioned engaging in social activities, such as calling loved ones or spending time with friends to keep themselves feeling well (35 or 68.6% of responses), but were significantly less likely to mention this when they were not feeling well (19 or 37.3% of students, *X*^*2*^ (1, 51) = 10.38, *p* = 0.002). Conversely, 12 (23.5%) students reported doing a solitary activity, e.g., “play guitar, watch tv and movies, reading” to keep themselves feeling well, while 22 (43.1%) students reported doing something alone, e.g., “sleep more, skip the gym, watch Netflix, meditate,” when they are not feeling well (*X*^*2*^ (1, 51) = 4.37, *p* = 0.036).

### Interview results

A total of 20 (12 female and 8 male) participants agreed to sit down for an in-person interview with a student investigator. Four of the participants were 1^st^ year students and 16 were 2^nd^ year students at MCASOM-AZ. All 20 participants who chose to sit down for the interview completed their interview by supplying a response to all 20 prompting questions. The length of each interview ranged between 25 and 40 min.

Thematic analysis revealed several agreed upon patterns in student responses, and specific representative quotes are provided in Table 2. Firstly, no one thought that wellness was “over-rated”. Most students alluded to the importance of balancing relationships, self-care, and academics in achieving wellness (the word “balance” was mentioned 65 times). Students largely agreed that wellness is contextual and inseparable from the broader culture of medical training. Given this, further discussion revealed a theme of subtle skepticism about addressing “wellness” in medical school without addressing a predominant medical culture that contributes to student and physician burnout and insufficiently emphasizes wellness. These medical students strongly discouraged mandatory wellness activities (e.g., “The only wellness activities I didn't enjoy were when we had them in the classroom, and it felt like they were teaching us how to be well…wellness is something that originates in you and it doesn't need to be so structured.”), but they were more open to student-initiated activities than school-initiated programs. Furthermore, many described the importance of a diverse and inclusive conceptualization of wellness that allows for individual differences, choice, and flexibility. Almost all valued having easily accessible school resources available such as mental health counselors and academic success advisors, some mentioned that they had utilized the services, and several said that while they would first seek support from family and friends before going to a counselor, they would utilize services if necessary. In terms of peer support, almost all mentioned the importance of friends and classmate support, though many found that their classmates also added to stress when they stressed out about school (“The ever-impending doom of like certain classmates talking about it all the time. What they're doing, and just that pressure to compare yourself to other people, which I tried to step away from.”). Many acknowledged that the pass/fail system decreased academic stress and made it easier to feel support and camaraderie from their peers. Also, several elaborated on why they are less social when stressed. (“When I'm feeling stressed, that's when I most feel like I need time to myself. Partly because the things that stress me out are often social interactions.”) Finally, all interviewees felt comfortable talking with KC, and many mentioned that they enjoyed the opportunity to talk confidentially to a peer and be listened to. These themes align with each other to reveal a broader consensus attitude around the nature of medical student wellness and opportunities to improve it.

**Table 2:** Predominant themes identified from sit-down interviews, with representative quotes

Predominant Theme	Representative Quote
**Students place value on wellness and have unique conceptions of wellness**	*“That could be spiritually, religiously, living a moral life in accordance with your morals. No cognitive dissonance in your life. You're living the life that you feel is right, and you're progressing towards your goals, be they career, family, personal, whatever”*
	*“For me, still a large part of my identity and how I view myself is in my abilities, my academics, and my desire to become a doctor. At the end of the day, I feel like I have multiple views in myself. Centrally, when I introduce myself to someone I say first and foremost I'm a medical student. If I was to focus on all of those other aspects of my wellness that's great, but if I didn't have a chance to actually become a doctor I think that would affect me a lot too.”*
	*“For me wellness honestly means that I get to have a balance in my life between the things I prioritize. It means not doing too much of one thing and letting that overtake my life.”*
	*“For me, a lot of my wellness stems from my interactions with my family and friends. That's the top thing, to maintain those relationships.”*
**Wellness is viewed as contextual and can’t be separated from medical culture**	*“You take that type of person who clearly doesn't value wellness. That's one party, and there's that subset that still exists to this day where that's what it takes to be a doctor. That's still trickled down into the idea of medicine…. systematic mentality coming down on us from both physicians and these ideas that have been ingrained in students”*
	*“It's one of those environments that with certain chronic illnesses and with certain external stressors like being in medical school. It's exacerbated environment”*
	*“Burnout has this connotation that it's a shortcoming on the part of the people experiencing burnout, rather than a shortcoming of the construct of the culture that people who are experiencing burnout are working in”*
**There is skepticism towards wellness efforts without addressing predominant medical culture**	*“We're gonna push our students really hard. We're gonna have high expectations. This is gonna be a rigorous curriculum. This is gonna stress them out, but it's OK because we have a wellness program… It’s treating the symptoms rather than the problem.”*
	*“The people who value wellness aren't rewarded by the system the way it's set up currently, which is ironic… yes you can incorporate wellness, but until the system rewards it, there's only so far you can take it.”*
	*“In an academic setting, it's [wellness] always an adjunct right? It's like, "We want all our students to perform well academically. Also, here's wellness over here," off to the side. I get frustrated because I feel like it's something that is talked about but not truly addressed.”*
	*“Keeping the exact same system, but also, now there's a yoga class you're supposed to go to, doesn't address the real problem.”*
**Mandatory wellness activities are discouraged**	*“I wonder about the efficacy of an institution almost wagging a finger and saying, "You should." Shoulds are a really good way to have people turn their backs on it.’*
	*“I'll say that I don't necessarily find mandatory wellness things I need to go to particularly helpful. Going back to my timing and business issues, it sounds like just another thing on the list. I find more intrinsically motivated wellness activities to be more helpful.”*
**Individual differences and preferences for wellness activities should be honored**	*“It's become grouped because that's what's been studied into a specific subset of ideas and activities, such as mindfulness, activities like yoga, tai chi, studying meditation, implicit understandings, and things like that”*
	*“It's the students that defines wellness as physical fitness, socialization and stuff like that that really benefit from the wellness program”*
	*“…there is a little bit of that barrier where I'm not fully convinced that you know where I'm coming from. Perhaps, maybe having student moderators would be a really good way to ease that, as well.”*

## Discussion

Key themes mutually supported by the questionnaire and interview responses included these pre-clinical students’ conceptions of wellness, the relationship between wellness and academic performance, and the impact of the atmosphere of medical education on wellness. Although unsurprising for this academically-minded study population, Academic Performance was second only to Family as an important factor in overall well-being. One student elaborated, “a large part of my identity and how I view myself is in my abilities, my academics, and my desire to become a doctor.” While important for well-being and central to identity, [academic] work was also cited by almost all as an obstacle to wellness. One participant commented, “school is often not the stressor, but rather what exacerbates other stressors,” and another referred to school as “the exacerbated environment.” This may explain why by the end of the academic year there is a 17.1% decrease in those who highly rank Academic Performance in their well-being.

Furthermore, results bifurcated into two groups. Students who ranked Academic Performance highly in well-being were more likely to choose these extrinsic achievements as important to success in medical school while those who lowly ranked Academic Performance were less likely. This bifurcation was reflected in the interview, as some students gauged their success by extrinsic measures (“a very metric ingrained definition of success”), and others gauged it more holistically (“I have some level of mastery of the material [while] not feeling the class taking over my life”). Several students mentioned that being top was not worth forfeiting wellness (“Even if I was getting the top score on an exam, if I was sacrificing sleep or friendships to do that, I wouldn't feel like that was a success. I would feel like that was not worth the price.”). Those who ranked Academic Performance highly for well-being were also less likely to rate 10/10 that maintaining wellness in medical school was important, as if wellness was contained within academic performance and did not require additional attention. To our knowledge, this dichotomy has not been shown in previous studies. Given that we found a decrease over time in the percentage who ranked academic performance as key to wellness, it may be that students began to shift priorities toward internal rewards versus external rewards. Better understanding these associations is an area ripe for more research.

At all three timepoints Family, Academic Performance, and Friends were consistently the highest ranked in their importance to overall wellness. These findings are thoroughly reflected in the interview responses, which allowed further exploration into the individual conceptions of wellness that are enabled by these factors. Throughout the interviews, when students were asked to elaborate their idea of wellness, mention of Family and Friends numerous times (70 mentions of family and 91 mentions of friends) and the role of Academic Performance is mentioned in enough detail to warrant separate discussion. Not a single interviewed participant mentioned Financial Stability as a factor contributing to their wellness, and only one person mentioned finances as one of the barriers to wellness. Although this finding may not be generalizable across school, geographic area, and/or year in schooling, the lower relative weight of this factor during preclinical years is supported by prior findings [20]. Of note, while many previous studies outline the various self-care behaviors employed by students, [16] interview and quantitative data revealed how these behaviors change depending on current stress level, specifically regarding the choice to socialize.

Through conversation, it could be seen that personal definitions of wellness were broad and deep. Nearly all participants included social, physical, emotional wellbeing into their definition, often explicitly citing the “health triangle,” but they also identified several factors that weighed particularly heavily for them as individuals but are not often discussed, like travel and romance. There was frequent conversation around spiritual well-being, and multiple students commented on their search for inner peace, quietude, and purpose. The importance of family featured prominently both for coping and academic success, suggesting that disruptions (e.g., death or illness of family member) could disproportionately negatively impact wellness and therefore warrants further study. Although one may expect that the relative influence of family might wane as immersion in the medical community and career path deepens, this was shown not to be the case according to the survey data, which continued to be highly favored during repeat midterm and final assessments and the interviews in which participants explained the value of family (e.g., “I'll call up my mom, my dad, my grandma, my brothers, sister, just talk to them and connect with them…I feel that helps keep me grounded. They give me perspective.”). Finally, the pass/fail system and the awareness of school resources (even if not used) were frequently mentioned as comforting. These factors may partly explain our previously reported outcomes in this same cohort [22] in which we observed decrease in perceived stress and increase in wellbeing over the course of the year, despite no difference between those who did or did not participate in wellness curriculum.

Seeing as academic work is a major factor of overall student wellness, it follows that student wellness was contextualized within the academic environment. Even early in their medical education, participants had clear opinions towards the atmosphere of medical education, commonly labeled “the system,” and regarded it as a hindrance to wellness. Students frequently commented on wellness curricula as insufficient and misguided, with four students referring to them as a “band-aid.” Another focused the issue on how “Wellness is often portrayed in only its negative form, burnout, which also has this connotation that it’s a shortcoming on the part of the people experiencing burnout rather than a shortcoming of the construct.” Altogether, a clear theme of skepticism emerged towards wellness efforts that fail to address the predominant medical culture. This sentiment was similar to the well-known aspect of medical training commonly referred to as the “hidden curriculum,” whereby trainees are placed in a predominant medical culture that models behaviors in the clinical workplace that are different or antithetical to what is explicitly taught [10, 39].

Given the close and conflicted relationship between wellness and academics as mutually elucidated through the survey and interview data, it follows that wellness curricula ought to be conceived alongside academic curricula and that academic work (cited universally as the obstacle to wellness activities) be made more manageable [18]. One student expressed “Maybe the answer is not necessarily to have more extra things, but maybe to structure the academic part of school in such a way that it's not as taxing.” Evidenced based examples of this include a pass/fail grading system, [12], “well-being day” absences, [19] and integrating wellness within existing coursework [40].

Both in their interview and free-text survey response, students preferred wellness programming that is student-led. These sentiments have been echoed elsewhere [14, 15]. Furthermore, we are seeing a national trend for students preferring to learn content individually and through mixed resources as opposed to traditional scheduled classroom didactics [41].

Just as wellness was found to be diversely conceptualized through our in-depth interviews, a wellness curriculum should not be narrowly biased towards conventional and stereotypic conceptions. Students described this bias for “people who prefer exercise and mindfulness” and as a narrow “subset of ideas and activities like yoga.” Instead, activities that are cerebral, or otherwise less stereotypically associated with wellness such as book club, special interest groups, or integrating humanities into core coursework may be more inclusive and well-received. As one student summarized, “One thing that I think is not emphasized enough in wellness is self-efficacy and locus of control.” Lastly, and consistent with the main direction of this study to focus on individual conceptions of wellness, many students identified a need for more reflection on the attitudes surrounding their personal wellness and see the value of such reflection. This research interview was noted by many participants as a welcomed occasion for such reflection, suggesting a potential role for peer coaching as a strategy to foster reflection and peer support.

The findings of this study were limited by small sample sizes for questionnaire responses at all three timepoints, as well as many dropouts at the midterm and final timepoints. This may introduce the bias that students who were feeling particularly unwell were excluded from later responses. Furthermore, study findings reflected only the experience of students in the preclinical years 1 and 2, where there is not yet the additional stressor and rewarding experience of hands-on patient care. Due to the small student body at the time, in order to ensure anonymity we chose not to collect detailed demographic information such as race/ethnicity, sexuality, religion, socioeconomic status, disability status, marital status, living situation, and medical school class. We did not have a control or comparison group by which to evaluate the responses. Additionally, although having a student interviewer may have allowed for greater trust from participants, it could have introduced an element of bias, whereby students may have felt the pressure to echo sentiments that they may have believed the interviewer or the greater student body may have held. Finally, when examining importance of factors, we did not ask participants to rate each factor and thus we don’t really know how important each factor was to the students. The surveys reported here were not empirically validated, although our results do cohere with findings from previously reported outcomes using empirically validated measures in the same sample [22].

## Conclusions

Mixed methods qualitative research can provide important insights to understand and explain the quantitative and categorical data. As predicted, medical students themselves hold a wealth of insight into what wellness means for them and how it enables their continued personal and professional success. These insights proved as diverse as the students themselves, while also revealing certain recurrent themes. A central theme was the conflicted relationship between wellness and academic performance, wherein academics is critical to student identity and their definition of wellness while also the largest barrier. Students diverged and fluctuated in how closely they perceived this relationship between academic performance and wellness. Another resounding theme was the emphasis placed on family and independent activities, especially when the intent is to recover well-being instead of maintaining it. Curricular improvements follow directly from these themes and student insight into the current atmosphere of medical education. Medical schools may find similar attitudes amongst their students and may thereby benefit from these curricular improvements, especially if implemented within a broader, organized effort as prescribed in prior literature [1] and at other medical schools [13, 18].

## Supplementary Information

**Additional file 1.** Questionaire 1.

## Data Availability

The datasets generated and/or analyzed during the current study are not publicly available due to the nature of qualitative data as audio files of specific interviewees who have not consented for their individual voices to be heard by parties aside from direct investigators. Quantitative data from survey results are outlined clearly in the manuscript but records from survey software used to gather data (REDCap) are available from the corresponding author on reasonable request.
