# Supplementary material for: Medical students describe their wellness and how to preserve it
Source: BMC Med Educ. 2022 Jun 28;22:510. doi: 10.1186/s12909-022-03552-y (PMC9241274; doi:10.1186/s12909-022-03552-y)
Supplement: Supplementary file 1 — Additional file 1. Questionaire 1. [file 12909_2022_3552_MOESM1_ESM.pdf]

## Questionnaire 1

Age
- ☐ ≤ 23
- ☐ 24-29
- ☐ ≥ 30

Gender
- ☐ Female
- ☐ Male
- ☐ Non-conforming

*Please rank the following in order of most to least important to your overall wellbeing (1 = most important)*

___ Academic performance
___ Physical fitness
___ Family
___ Financial stability
___ Friends
___ Hobbies
___ Alone time
___ Sleep

*What do you need to accomplish in order to feel successful as a medical student? Please rank the following in order of most to least important to your success ( 1 = most important)*

___ Do well on board exams
___ Maintain physical fitness
___ Publish research
___ Maintain a healthy diet
___ Keep up with friends
___ Become involved in extracurriculars (e.g. interest groups, committees)
___ Match into first choice residency program
___ Spend time with family

*On a scale from 1 to 10, how important is it to you to maintain your wellness while in medical school?* _________
*(1 = not important, 10 = most important)*

*What do you do to keep yourself feeling well?*

________________________
________________________
________________________
________________________

*What do you do when you are not feeling well?*

________________________
________________________
________________________
________________________

*What gets in the way of you doing those things? Please check all that apply.*

- ☐ School work
- ☐ Family responsibilities
- ☐ Research
- ☐ Friends and other social responsibilities
- ☐ Finances
- ☐ Physical or mental health
